# Supplementary material for: Cooling low-dimensional electron systems into the microkelvin regime
Source: Nat Commun. 2022 Feb 3;13:667. doi: 10.1038/s41467-022-28222-x (PMC8814190; doi:10.1038/s41467-022-28222-x)
Supplement: Supplementary file 1 — Supplementary Information [file 41467_2022_28222_MOESM1_ESM.pdf]

Cooling Low-Dimensional Electron Systems into the Microkelvin Regime
Supplementary Information

Lev V. Levitin,[1,*] Harriet van der Vliet,[1,†] Terje Theisen,[1] Stefanos Dimitriadis,[1,‡] Marijn Lucas,[1] Antonio D. Corcoles,[1,§] Ján Nyéki,[1] Andrew J. Casey,[1] Graham Creeth,[2,¶] Ian Farrer,[3,**] David A. Ritchie,[3] James T. Nicholls,[1] and John Saunders[1]

[1] *Department of Physics, Royal Holloway, University of London, Egham TW20 0EX, UK*
[2] *London Centre for Nanotechnology, University College London, London WC1H 0AH, UK*
[3] *Cavendish Laboratory, University of Cambridge, JJ Thomson Avenue, Cambridge CB3 0HE, UK*

(Dated: November 22, 2021)

FURTHER DETAILS OF THE EXPERIMENTAL SETUP

All experiments were conducted on a home-made copper nuclear demagnetisation stage, precooled by a custom-built "wet" Oxford Instruments Kelvinox 400 dilution refrigerator. The metal cell was constructed out of OFHC copper and high purity silver, with small amounts of niobium, copper-nickel, silicon and amorphous dielectrics, such as PEEK, Teflon, Stycast 1266, paper and GE varnish. The heat exchangers were fabricated from 70 nm silver powder. Following Ref. [32] silver epoxy filters, Fig. 2d, were made using Epotek E4110 conducting epoxy. This epoxy was also used to attach the 4 mm×4 mm 2DEG device to a copper holder that was screwed to the immersion cell lid, to ensure good thermalisation of phonons in GaAs. The plastic cell was made out of Stycast 1266 epoxy and included the materials listed above.

Figure S1 shows the 4.2 K attenuation characteristics of the low-pass filters used in this work.

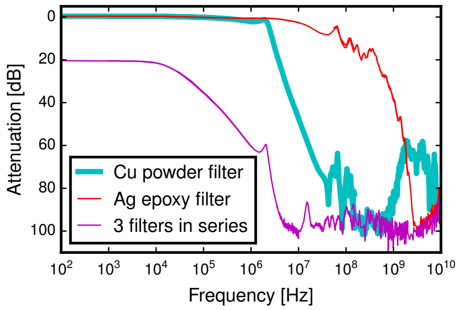

Figure S1. Attenuation characteristics of the different low-pass filters at 4.2 K. The Cu powder filter, shown in Fig. 2c, transmits near 100 MHz and 3 GHz, above its 2 MHz cut-off frequency. The Ag epoxy filter with a single superconducting core, see Fig. 2d, has a 100 MHz cut-off, higher than in Ref. [32] due to the lack of dissipation in the superconducting core. The multi-line Ag epoxy filter used between the 2DEG NT and its SQUID sensor exhibits similar attenuation for the common mode signals. The differential mode, which has not been characterised, may show less attenuation. A chain of 3 filters: single-line Cu powder and Ag epoxy filters plus an $R = 500\,\Omega$, $C = 20\,\mathrm{nF}$ filter (a pair of Fig. 2b filters wired in parallel, such pairs were used in the NT heater lines to reduce mixing chamber heating when driving large currents through the heater), has a 16 kHz cut-off determined by the RC filter and at least 100 dB attenuation at 0.03-3 GHz. The "lossy coaxial" design of the Ag epoxy filters [61] and the heterogeneity of the chain components makes the transmission above the cut-off frequency, as exhibited by the Cu powder filter, unlikely. None of the filters match the 50 Ω impedance of the spectrum analyser and vector network analyser used in this characterisation. This mismatch potentially results in an overestimated attenuation.

NT HEATER RESISTANCE

The NT heater resistance, which determines the magnitude of $\dot{Q}^{\mathrm{J}}_{\mathrm{NT}}$, could not be measured in situ, and was inferred from $R(l)$ measurements of similar wires of different lengths l shown in Fig. S2.

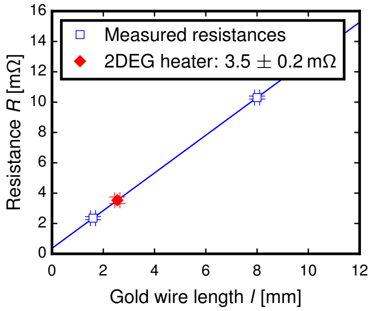

Figure S2. The resistance of the 2DEG NT heater is inferred from its length from $R(l)$ measurements on similar wires of different lengths, all connected with wedge-bonded superconducting aluminium wires. The non-zero intercept in the $R = a + bl$ fit is attributed to the gold-aluminium contact resistance. Error bars represent s.d.

2DEG RESISTANCE MEASUREMENTS

In addition to the filtering shown in Fig. 2, extra RC filters were connected to I+ and I− lines in the metal cell to provide independent current and voltage lines, as shown in Fig. S3a. Therefore the four-terminal resistance

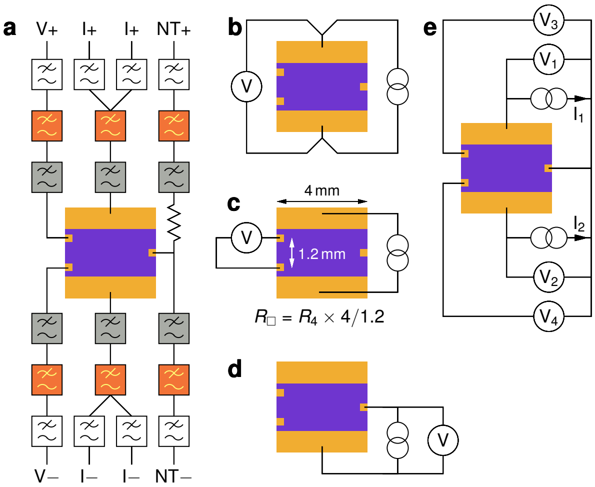

Figure S3. Details of the electrical measurements. **a** Electrical connections to the 4 mm×4 mm 2DEG device in the metal cell, allowing 4-terminal resistance measurements using the NT and I± ohmic contacts. **b-d** Measurements of $R_{\mathrm{I+\,to\,I-}}$ (**b**), $R_\square$ (**c**) and $R_{\mathrm{NT\,to\,I-}}$ (**d**). The 2DEG sheet resistance $R_\square$ was obtained from the measured 4-terminal resistance R_4 by simple geometrical scaling. **e** Measurement of $R_{\mathrm{NT\,to\,IV}}$. Currents I_1 and I_2 fed into the I± ohmics were adjusted to equalise their potentials V_1 and V_2, thus giving the resistance from the NT to the I± ohmic contacts to be $R_{\mathrm{NT\,to\,I\pm}} = V_1/(I_1 + I_2)$. Simultaneously $V_3 \approx V_4 \approx 0.99V_1$, demonstrating that $R_{\mathrm{NT\,to\,IV}} \approx R_{\mathrm{NT\,to\,I\pm}}$.

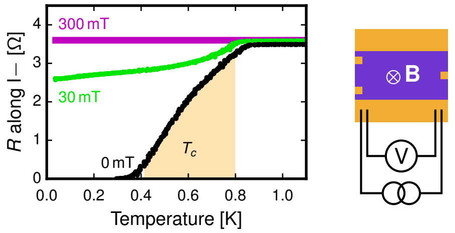

Figure S4. Superconducting behaviour in the I− ohmic contact (left), showing the suppression of T_c when a magnetic field **B** is applied perpendicular to the 2DEG. These resistance measurements along the top of the contact were performed on the 4 mm×4 mm device on a separate cool-down using a different wire bond configuration (right).

measurements that involve I± contacts, such as $R_{\mathrm{NT\,to\,I-}}$, include Ag epoxy and Cu powder filters and the interconnecting Cu and NbTi wires, contributing less than 0.1 Ω. Figure S3e shows that a measurement of $R_{\mathrm{NT\,to\,IV}}$ does not require shorting the I± and V± ohmics. Signatures of superconductivity in the I− ohmic contact are shown in Fig. S4. Table S1 summarises all the relevant low temperature resistances.

A different sample of identical design, also fabricated using wafer W476, was investigated in the plastic cell. The LED was situated outside the cell and the light had to travel through the cell wall, leading to less efficient

Table S1. Low temperature resistances in the 4 mm×4 mm device used in the metal cell. The resistance $R_{\mathrm{I_+\,to\,I_-}}$ determines the magnitude of the Joule heating $\dot{Q}_\mathrm{e}^\mathrm{J}$. The resistance from the NT ohmic contact to all other contacts connected in parallel, $R_{\mathrm{NT\,to\,IV}}$, determines the thermal conductance G_e^WF predicted from the WF law.

	Before illumination	After illumination
$R_\square$	32.5 Ω	6.4 Ω
$R_{\mathrm{I+\,to\,I-}}$	16.8 Ω	4.4 Ω
$R_{\mathrm{NT\,to\,I+}}$	31.0 Ω	7.7 Ω
$R_{\mathrm{NT\,to\,I-}}$	31.4 Ω	7.7 Ω
$R_{\mathrm{NT\,to\,IV}}$	26.8 Ω	6.1 Ω

Table S2. Low temperature resistances in the 2DEG device used in the plastic cell.

	Before illumination	After illumination
$R_\square$	48.0 Ω	15.3 Ω
$R_{\mathrm{I+\,to\,I-}}$	22 ± 1 Ω	7 ± 1 Ω

illumination, see Table S2, than in the metal cell. Single I± connections increased the uncertainty in $R_{\mathrm{I+\,to\,I-}}$. The absence of NT± lines made in-situ measurements involving the NT ohmic contact impossible.

^{3}HE TEMPERATURE

The plastic immersion cell was equipped with a ^{3}He noise thermometer, see Fig. S5. A 25 μm Au wire was wedge-bonded between two sintered 1 mm Ag wires. The whole assembly was enclosed in a leak-tight Stycast 1266 appendix cell, connected to the main cell with a capillary and surrounded with a Nb shield. The temperature T_bath of this composite Au+Ag resistor was read out with a

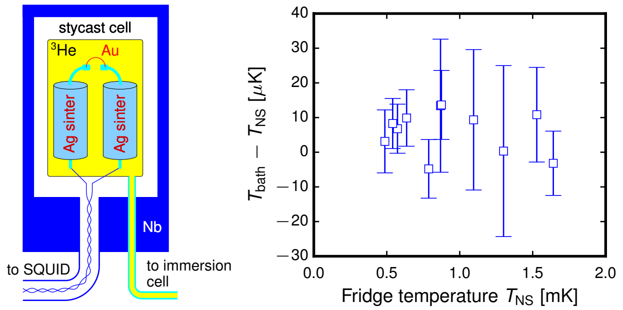

Figure S5. Thermalisation of ^{3}He. In the plastic cell T_bath was measured directly using a ^{3}He noise thermometer appendix (left). It agrees with the noise thermometer monitoring the nuclear stage temperature T_NS to within 20 μK (right). Error bars represent s.e.m.

SQUID current sensor. It was found to be within 20 μK of the nuclear stage temperature $T_{\rm NS}$ down to 0.5 mK, see Fig. S5.

A similar thermometer was not implemented in the metal cell, to avoid the construction overheads associated with bringing an additional twisted pair into the shielded environment. The low-heat leak nature of this environment plus significantly reduced amount of amorphous dielectrics in contact with ^{3}He, in comparison with the plastic cell, allow us to conclude that the thermalisation of the ^{3}He bath in this metal cell was at least as good as in the plastic cell. Therefore the temperature of the nuclear stage, monitored in the experiment with the metal cell, gives an accurate measure of the ^{3}He bath temperature $T_{\rm bath}$.

2DEG NOISE THERMOMETER CORRECTION

The sintered silver heat sinks at $T_{\rm bath}$, see Fig. 2, installed to reduce $\dot{Q}^0_{\rm NT}$, contribute $\delta = 5\%$ of the 2DEG NT resistance R, see Fig. S6. The measured Johnson voltage noise power spectral density

$$S_V = 4k_B(1-\delta)RT_{\rm NT} + 4k_B\delta RT_{\rm bath} = 4k_BRT^0_{\rm NT} \quad \text{(S1)}$$

gives the raw NT reading $T^0_{\rm NT}$, allowing us to infer the temperature $T_{\rm NT}$ of the NT gold wire

$$T_{\rm NT} = \frac{T^0_{\rm NT} - \delta T_{\rm bath}}{1-\delta}. \quad \text{(S2)}$$

The sub-20 μK gradient between the ^{3}He bath temperature and the measured nuclear stage temperature down to 0.3 mK results in a negligible 1 μK error in $T_{\rm NT}$.

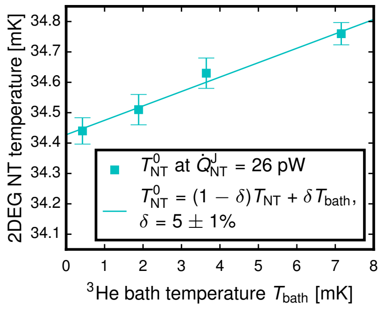

Figure S6. The fraction δ of the 2DEG NT resistance sitting at the ^{3}He bath temperature $T_{\rm bath}$. Here we present the raw 2DEG NT readings $T^0_{\rm NT}$ versus $T_{\rm bath}$ when high power is applied to the NT heater, such that the temperature $T_{\rm NT}$ of the NT gold wire is independent of $T_{\rm bath}$. A fit based on Eq. (S1) allows us to determine δ. Error bars represent s.d.

Table S3. Summary of the fit parameters in Eqs. (1), (2), (S3), (S5), and (S7).

	**Before illumination**	**After illumination**
R	59 Ω	27 Ω
α	0.24	0.33
K_0	7.0×10^{-12} W/K	
K_0'	1.6×10^{-12} W/K	
K_2	2.7×10^{-7} W/K^3	
K_3	4.9×10^{-5} W/K^4	
$K_{3.2}$	1.8×10^{-3} W/K$^{4.2}$	
X_4	1.1×10^{-2} W/K^5	

ADDITIONAL THERMAL TRANSPORT AND HEAT LEAK MEASUREMENTS

Figure S7 illustrates measurements of thermal conductances and heat leaks in the preliminary and auxiliary immersion cell runs. In contrast to the main experimental run, referred to below as M, in all of these measurements the immersion cells were filled with pure ^{3}He, resulting in 2D solid ^{3}He adsorbed on all surfaces inside the cells and consequently increased $G_{\rm He}$ [48]. For each NT and NT heater the NT correction factor δ and heater resistance were obtained using procedures described above. The coefficients in thermal conductance models obtained in the fits are summarised in Table S3.

Run A. A 4 mm×4 mm 2DEG device was cooled in the plastic immersion cell with Cu powder filters. The sample was damaged during disassembly after the run, and so a second sample from wafer W476 and with similar electrical characteristics was used in the metal cell. Figure S7a shows the non-local thermal conductance measured between 7 and 30 mK, which was found to follow

$$G^{\rm A}_{\rm N}(T) = K_{3.2}T^{3.2} \quad \text{(S3)}$$

behaviour that is consistent with a sum of T^3 and T^4 terms (see Eqs. (2) and (S5)) observed in runs M and B. Unlike those runs, $G^{\rm A}_{\rm N}(T)$ is independent of the illumination, which may reflect a more favourable layout of the gold wire bonds to the I± ohmics in run A. The heat leak was inferred from measurements of $T_{\rm NT}$ vs $T_{\rm bath}$ at $\dot{Q}^{\rm J}_{\rm e} = 0$, Fig. S7d, and integrating Eq. (S3)

$$\dot{Q}^0_{\rm e} = \int_{T_{\rm bath}}^{T_{\rm NT}} G^{\rm A}_{\rm N}(T)\,dT. \quad \text{(S4)}$$

Equation (S4) gave consistent results above $T_{\rm bath} = 4$ mK. This was not the case at lower temperatures suggesting that $G^{\rm A}_{\rm N}(T)$ does not extrapolate to low temperatures according to Eq. (S3). Therefore the data at $T_{\rm bath} < 4$ mK, shown in grey in Fig. S7d, were excluded

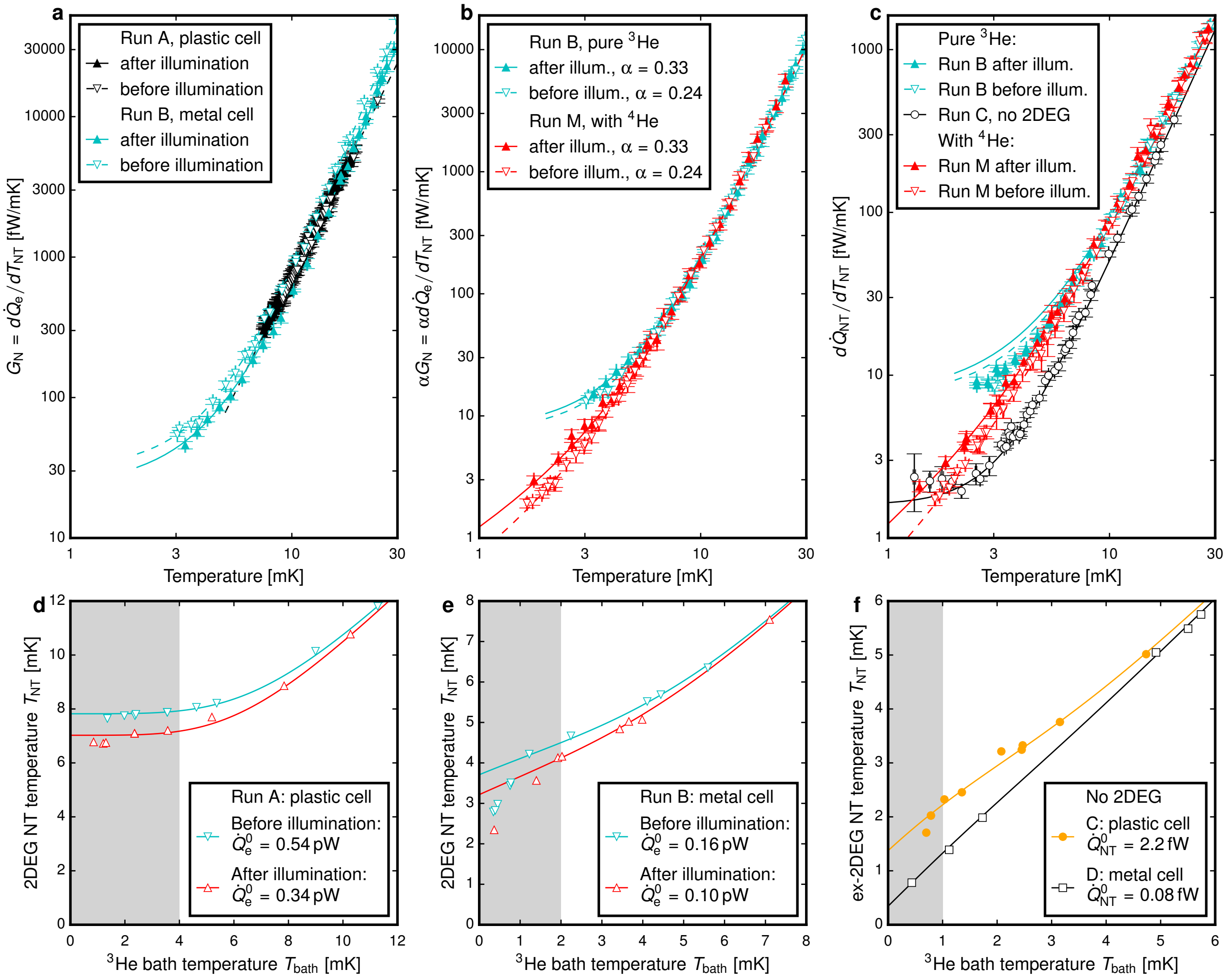

Figure S7. Thermal conductances and heat leaks in preliminary and auxiliary experiments. **a** Non-local thermal conductance G_N measured on two different 2DEG devices in the preliminary runs A and B, both with pure ^{3}He. The data are described by Eqs. (S3) and (S5). **b** G_N measured on the same 2DEG device in the metal cell during runs B and M, described by Eqs. (S5) and (2). The data are shown scaled by α measured in run M. **c** Response of 2DEG NT to NT heater in runs B, C and M. In run M it provides a measurement of $G_\Sigma(T_{NT})$, Eq. (1), however in run B the measured $d\dot{Q}_{NT}/T_{NT}$ (cyan triangles) deviate from the predicted behaviour, see Eq. (S6) (cyan lines) below 7 mK, attributed to significant $\dot{Q}_e^0$. The Kapitza conductance between the NT assembly and ^{3}He measured in run C is described by Eq. (S7). **d-e** Heat leak to 2DEG measured in runs A and B evaluated from G_N in **a**. **f** Heat leak to NT + gold thermal link + NT heater in run C and to NT alone in run D. The grey areas in **d-f** indicate the low temperature regime into which $G_N(T)$ and $G_{He}(T)$ can not be reliably extrapolated. These were excluded from the heat leak evaluation. Error bars in **a-c** represent s.d.

from the determination of $\dot{Q}_e^0$. Due to the lack of NT heater G_Σ and α could not be measured directly, so T_e in Fig. 1c was roughly estimated using the parameters derived for the second 2DEG device, with the heat leak $\dot{Q}_e^0$ being the only input from Run A.

Run B. The second 2DEG device was cooled in the metal cell with Cu powder filters. In this run G_N was found to follow

$$G_N^B(T) = G_N^M(T) + K_0/\alpha, \tag{S5}$$

see Fig. S7a, with $G_N^M(T)$ and α obtained in run M, Eq. (2). This corresponds to the thermal conductance between the NT and the ^{3}He bath

$$G_\Sigma^B(T) = G_\Sigma^M(T) + K_0, \tag{S6}$$

with $G_\Sigma^M(T)$ given by Eq. (1). Here K_0 represents an extra channel in G_{He} due to the nuclear magnetism of 2D solid ^{3}He absorbed on the noise thermometer assembly when the cell is filled with pure ^{3}He in run B, but suppressed by ^{4}He plating in run M [48]. The discrepancy between $d\dot{Q}_{NT}/dT_{NT}$ measured in run B and Eq. (S6) below 7 mK, see Fig. S7c, is attributed to the breakdown of the differential thermal conductance measurement $d\dot{Q}_{NT}/dT_{NT}$ in the presence of significant $\dot{Q}_e^0$,

in the regime where G_Σ/G_N is temperature-dependent. The heat leak, Fig. S7e, was measured by the same procedure as in run A and the extrapolation of the measured $G^\mathrm{B}_\mathrm{N}(T)$ was found to hold down to 2 mK.

Run C. To measure G_He separately from G_e, an NT + gold thermal link + NT heater assembly was installed in the plastic cell, similar to that used in the metal cell, but with no 2DEG and with the NT ohmic contact substituted with a gold bonding pad on a silicon chip. The total length of the NT + thermal link + heater gold wire chain was similar to the metal cell, with a surface area of 0.8 mm^2. Figure S7c shows the Kapitza conductance between this gold wire and ^{3}He that follows

$$G^\mathrm{C}_\mathrm{He}(T) = K_0' + K_3T^3, \tag{S7}$$

with the same K_3 as in Eq. (1), but without the K_2T^2 term and with K_0' smaller than K_0 observed run B, Eq. (S5). We attribute this discrepancy to the strong thermal link between the 0.04 mm^2 surface of the NT ohmic contact and ^{3}He, potentially related to the coupling between ^{3}He nuclear spins and magnetic Ni in the ohmic contact. The heat leak, Fig. S7f was obtained as

$$\dot{Q}^0_\mathrm{NT} = \int_{T_\mathrm{bath}}^{T_\mathrm{NT}} G^\mathrm{C}_\mathrm{He}(T)\,dT. \tag{S8}$$

Run C provides the upper bound on $\dot{Q}^0_\mathrm{NT}$ for runs A and B and justifies ignoring the small $\dot{Q}^0_\mathrm{NT}$ when evaluating much larger $\dot{Q}^0_\mathrm{e}$.

Run D. After the end of run M, the gold thermal link between NT ohmic and NT was replaced with aluminium. Isolating the NT from the 2DEG and NT heater, allowed us to measure just the heat leak into the 1.6 mm long NT, as opposed to run C, where the combined heat leak into 10 mm long chain of gold wires was measured. To obtain the heat leak, Fig. S7f, we scale G^C_He in Eq. (S8) by the wire length. This provides the lower bound on $\dot{Q}^0_\mathrm{NT}$ for run M, and allows us to obtain an upper bound on $\dot{Q}^0_\mathrm{e}$ and T_e, which is the main purpose of our thermal model.

POTENTIAL IMPROVEMENTS IN COOLING

Here we consider the cooling of the 2DEG device characterised in the metal cell in the absence of the superconductivity in the ohmics, while retaining all other parameters. We expect to obtain such conditions when a magnetic field of 0.1 T is applied parallel to the plane of the 2DEG [46]. With the I± ohmics fully contributing to the cooling of the 2DEG, they dominate G_IV, Fig. 3g, therefore $L_0T/G_\mathrm{IV} = \alpha R_\mathrm{e} = R_{\mathrm{I+\,to\,I-}}/4$ (two halves of $R_{\mathrm{I+\,to\,I-}}$ connected in parallel). Using the resistances measured at 1.0 K, above T_c, $R_\mathrm{e} = R_{\mathrm{NT\,to\,IV}}(1.0\,\mathrm{K}) = 27\,\Omega$ ($7\,\Omega$), $\alpha = 0.16$ (0.18) before (after) illumination, as measured above T_c. Then for $T_\mathrm{bath} = 0.3$ mK and $\dot{Q}^0_\mathrm{e} = 0.7$ fW (1.8 fW) Eqs. (3) and (4) give $T_\mathrm{e} = 0.6$ mK (0.5 mK). By optimising the fridge and heat exchanger performance we expect a bath temperature of $T_\mathrm{bath} = 0.1$ mK and an electron temperature in the 2DEG of $T_\mathrm{e} = 0.5$ mK (0.4 mK).

If the superconductivity is to be tolerated, an overlayer of evaporated gold or a network of gold wirebonds can improve the effectiveness of the large I± contacts at least to the level of the small NT ohmic. By scaling the effective contact resistance of the latter $R^*_\mathrm{NT} = (1-\alpha)R_\mathrm{e}^{\text{after illum.}} = 18\,\Omega$ with the perimeter we get $R^*_{\mathrm{I\pm}} = 3\,\Omega$ for each I+ and I−. As these ohmics now dominate the smaller V±, we estimate $G_\mathrm{IV} = L_0T/\big(R_{\mathrm{I+\,to\,I-}}/4 + R^*_{\mathrm{I\pm}}/2\big)$. Assuming that the extra gold does not change the small NT ohmic significantly, we take G_NT to be unchanged by this procedure. This gives $R_\mathrm{e} = 50\,\Omega$ ($21\,\Omega$), $\alpha = 0.11$ (0.12) before (after) illumination leading to $T_\mathrm{e} = 0.6$ mK at $\dot{Q}^0_\mathrm{e} = 0.7$ fW (1.8 fW) and $T_\mathrm{bath} = 0.1$ mK in both cases.

* l.v.levitin@rhul.ac.uk
† Now at Oxford Instruments Nanoscience, Abingdon, Oxfordshire OX13 5QX, UK.
‡ Now at Department of Physics, Imperial College London, London SW7 2AZ, UK.
§ Now at Thomas J. Watson Research Center, Yorktown Heights, NY 10598, USA.
¶ Now at Praesto Consulting, Dublin, D02 A342, Ireland.
** Now at Department of Electronic and Electrical Engineering, University of Sheffield, Sheffield S1 3JD, UK.

[32] Scheller, C. P. *et al.* Silver-epoxy microwave filters and thermalizers for millikelvin experiments. *Appl. Phys. Lett.* **104**, 211106 (2014). URL https://doi.org/10.1063/1.4880099.
[46] Beauchamp, C. B. *et al.* Superconductivity in AuNiGe ohmic contacts to a GaAs-based high mobility two-dimensional electron gas. *Appl. Phys. Lett.* **117**, 162104 (2020). URL https://doi.org/10.1063/5.0028217.
[48] Hu, Y., Stecher, G. J., Gramila, T. J. & Richardson, R. C. Magnetic coupling in thermal-boundary resistance between thin silver films and liquid ^{3}He in the millikelvin regime. *Phys. Rev. B* **54**, R9639–R9642 (1996). URL https://link.aps.org/doi/10.1103/PhysRevB.54.R9639.
[61] Tancredi, G., Schmidlin, S. & Meeson, P. J. Cryogenic coaxial microwave filters. *Rev. Sci. Inst.* **85**, 026104 (2014). URL https://doi.org/10.1063/1.4863881.
